# Crown Ether-Capped Gold Nanoclusters as a Multimodal Platform for Bioimaging

**DOI:** 10.1021/acsomega.3c00426

**Published:** 2023-03-13

**Authors:** Patryk Obstarczyk, Anna Pniakowska, Nonappa, Marcin P. Grzelczak, Joanna Olesiak-Bańska

**Affiliations:** † Institute of Advanced Materials, Wroclaw University of Science and Technology, 50-370 Wrocław, Poland; ‡ Faculty of Engineering and Natural Sciences, Tampere University, FI-33720 Tampere, Finland

## Abstract

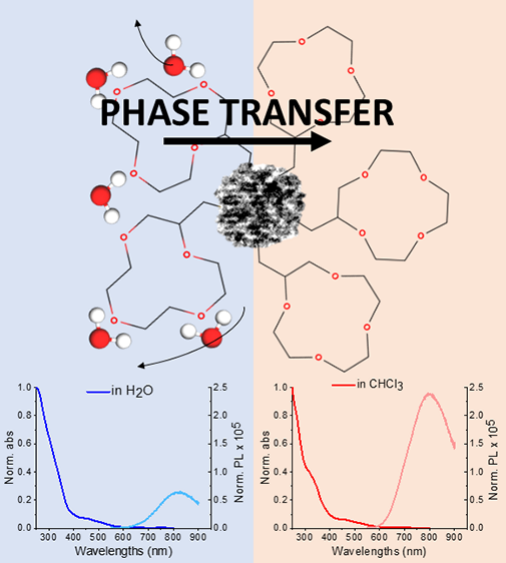

The distinct polarity of biomolecule surfaces plays a pivotal role in their biochemistry and functions as it is involved in numerous processes, such as folding, aggregation, or denaturation. Therefore, there is a need to image both hydrophilic and hydrophobic bio-interfaces with markers of distinct responses to hydrophobic and hydrophilic environments. In this work, we present a synthesis, characterization, and application of ultrasmall gold nanoclusters capped with a 12-crown-4 ligand. The nanoclusters present an amphiphilic character and can be successfully transferred between aqueous and organic solvents and have their physicochemical integrity retained. They can serve as probes for multimodal bioimaging with light (as they emit near-infrared luminescence) and electron microscopy (due to the high electron density of gold). In this work, we used protein superstructures, namely, amyloid spherulites, as a hydrophobic surface model and individual amyloid fibrils with a mixed hydrophobicity profile. Our nanoclusters spontaneously stained densely packed amyloid spherulites as observed under fluorescence microscopy, which is limited for hydrophilic markers. Moreover, our clusters revealed structural features of individual amyloid fibrils at a nanoscale as observed under a transmission electron microscope. We show the potential of crown ether-capped gold nanoclusters in multimodal structural characterization of bio-interfaces where the amphiphilic character of the supramolecular ligand is required.

## Introduction

The design of smart probes that are sensitive to biomolecules’ polarity is highly demanded^1,2^ in chemical as well as biological non-covalent effects (i.e., hydrogen bonding, bipolarity, hydration, and polarizability) as it plays a vital role.^3^ Much evidence indicates that the surface polarity is tightly involved in basic physiological processes, including protein denaturation and folding, membrane fusion, and enzymatic activity.^4^ Fluorescent imaging, due to its sensitivity, temporal as well as spatial resolution, and non-invasiveness, has become a primarily used research tool in biomedical science, being especially potent for in vivo studies.^5^ Conventionally, this method relies on small molecular probes of the distinct fluorescence arising from structural frameworks containing electron-donating and electron-accepting chemical groups. As imaging of biological samples relies on light penetration, in order to reach deep-tissue penetration, fluorescent probes should emit in the so-called optical window (NIR-1 located at wavelengths of 700–1000 nm).^6,7^ However, majority of reported probes based on organic dye architectures display suboptimal selectivity to polarity, moderate physicochemical merits (e.g., photostability, binding modes, in vivo residence time, and water solubility), and a lack of the modulation of probe hydrophobicity, which hinders their utility in bioimaging.^1,3^ Usually, to achieve high optical merits, the organic dye design is based on prolonged π conjugation, which may ultimately limit its binding affinity to bio-interfaces.^8^ Additionally, in order to perform detailed structural characterization on a sub-micron and nanoscale, fluorescence imaging has to be followed by, for example, electron microscopy imaging. However, organic probes characterized by fluorescence in the near-infrared (NIR) range of wavelengths and a high electron density (for cross-platform imaging) are challenging to design.

Recently, ultrasmall and thiol-protected gold nanoparticles, that is, gold nanoclusters (GNCs), have gained popularity as robust luminescent nanomaterials with relatively low toxicity and tunable optical and chemical properties for bioimaging applications.^9−12^ GNCs are a versatile group of ultrasmall (with a diameter below 2 nm) nanomaterials with potential applications in one-photon and two-photon imaging as their optical properties prevail those of standard dyes: they exhibit tunable (from UV to NIR) photoluminescence, large Stokes shifts (that can exceed 100 nm), and high photostability.^13−16^ Moreover, GNCs are sensitive to a single noble-metal atom change as their molecule-like UV–vis spectra may be easily tuned by the cluster core composition and protecting ligand type.^17^ Additionally, the physicochemical properties of GNCs governing the efficiency of their binding to biological materials can be easily tailored by the surface ligand functionality.^9,10,18^

GNCs can also be effective probes for electron microscopy. Applications of GNCs in structural studies of biological materials were presented in the work of Martikainen et al. where hydrophobic pockets of selected enteroviruses (EV1) were visualized using conjugates of gold nanoclusters with 102 gold atoms (Au_102_) with a fluorescent dye and selective target agent, the WIN compound.^19^ Virus hydrophobic pockets stained with GNCs were easily visualized with TEM. Gold nanoclusters were also applied in cryo-TEM imaging of a protein structure. Jagota et al. presented that Au_144_ GNCs attached to a protein complex allowed them to recover 3D coordinates of the protein structure via a computational method for cryo-TEM tilt-pair image analysis.^20^ The studies revealed the range of motion within the protein complex and proved the importance of GNCs in the rapid and cost-effective imaging and evaluation of the structure and molecular motion of proteins. One should note that, although nanoclusters of >100 gold atoms are characterized by a higher electron density, they usually exhibit no luminescence.^21^ Thus, for multimodal imaging, it is necessary to functionalize them with fluorescent molecules.^19^

Herein, we present 12-crown-4-SH-capped ultrasmall (∼1.78 nm) gold nanoclusters with amphiphilic properties that can be readily and reversibly transferred between aqueous and organic solvents. Crown ethers (CEs) are macrocyclic compounds successfully exploited in liquid–liquid and solid–liquid phase-transfer reactions as supramolecular molecules.^22^ The oxygen atoms present in the ring are capable of forming hydrogen bonds with water molecules that form a solvated shell, and the organically soluble ethylene oxide molecules give them amphiphilic properties.^23^ Crown ethers were also extensively applied in ion extraction,^24^ and their physicochemical properties are of great importance for phase-transfer catalysis^25^ or in studies on lipid membranes.^26^ Thiolated crown ether (18-crown-6) was previously used as a stabilizing ligand in the synthesis of bigger plasmonic gold nanoparticles^27−30^ where the nanoparticles’ hydrophobicity was modulated by complexation of alkali metals such as sodium and potassium and alkali-earth metal barium. Baghdasaryan et al. presented a successful ligand exchange between a Au_25_(2-PET)_24_ cluster and functionalized thiolated 18-crown-6.^31^ According to MALDI-TOF mass analysis, they obtained few exchange species (*x* = 5, Au_25_(2PET)_18–2*x*_(*CE*)_*x*_). ATR-FT-IR studies showed significant red shifts of C–O stretching vibration modes due to the metal ion (e.g., K^+^, Ba^2+^, and Eu^3+^) encapsulation into a crown cavity. However, the amphiphilicity of such a system (composed of mixed hydrophobic and amphiphilic ligands) was not investigated.

In our studies, we chose 12-crown-4 ethers as supramolecular capping ligands for GNCs. Among numerous crown-ether systems, the 12-crown-4 ether does not influence the homeostasis of the physiological ions such as K^+^ and Na^+^ as it is selective solely to Li^+^ cations.^32^ Moreover, it can cross the blood–brain barrier and form complexes with charged amino acids/hydrogen-bond networks or interact electrostatically with protein aggregates as presented for amyloid-β (Aβ).^33,34^ We present the synthesis of 12-crown-4 ether-stabilized GNCs and show that the ligand conformation is altering upon interactions with a protic environment, which modulates the entire electronic structure of GNCs (as seen in UV–vis spectroscopy and Fourier-transform infrared spectroscopy). Our GNCs exhibit NIR fluorescence and simultaneously present contrasting efficiencies under TEM.

In order to exploit the bioimaging potential of our GNCs, we applied them to image amyloids, which are protein aggregates organized due to the interplay between hydrophobic and hydrophilic interactions of peptides or proteins.^35,36^ Amyloids are the hallmark of numerous neurogenerative disorders, including Parkinson’s and Alzheimer’s diseases (PD and AD, respectively). They exhibit high morphological polymorphism governing their toxicity.^37,38^ Moreover, self-assembly of amyloidogenic peptides and proteins can lead to the formation of superstructures, namely, spherulites, which are heterogeneous at nano- and microscales.^39,40^ Spherulites are densely packed spherical superstructures with diameters varying from 5 to 150 μm and composed of unstructured cores and radially growing fibrils.^41^ They are characterized by the presence of numerous intermediate states of amyloid aggregates, including mature fibrils.^42^ Numerous studies indicated that distinct amyloid fibril morphologies may be more pathogenic than others.^38,43^ Therefore, rapid structural characterization of amyloids is clinically relevant for diagnosis, classification, and, in further perspective, the treatment of amyloid diseases. We showed that crown-ether-capped GNCs readily stain amyloids in the form of individual fibrils as well as amyloid superstructures—spherulites, as visualized under fluorescence and transmission electron microscopy. Hence, they are proven to be multimodal probes, which reproduce and reveal structural features of amyloid aggregates at micro- and nanoscales upon the staining procedure performed post amyloid incubation.

## Results and Discussion

**Figure 1:**
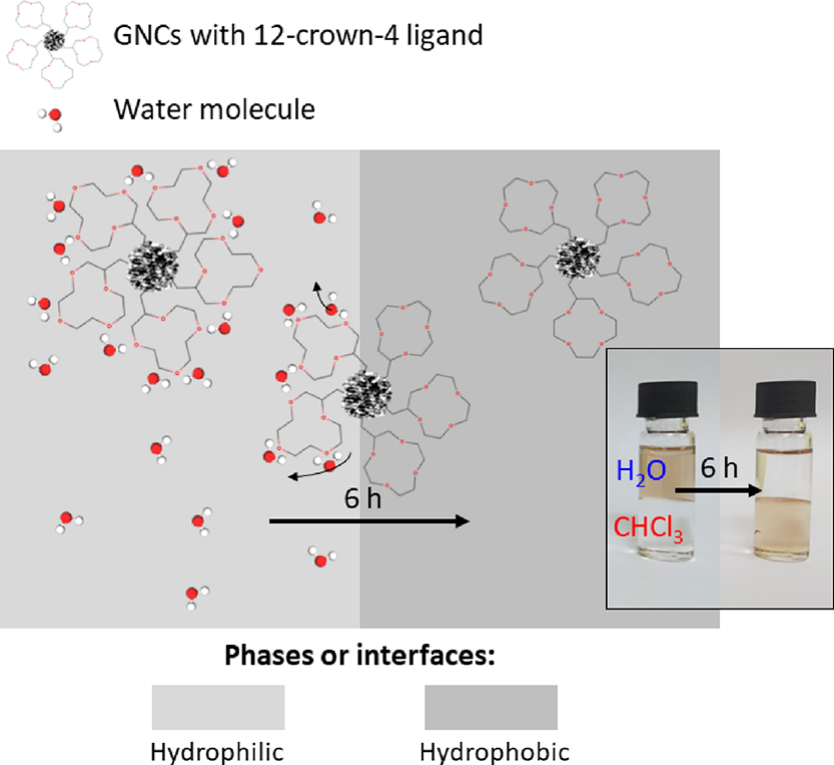
Schematic representation of GNCs capped with a 12-crown-4 ether and its amphiphilic properties—phase transfer from a hydrophilic to hydrophobic medium. Water molecules solvating crown-ether ligands are released upon interaction with the hydrophobic phase or interface, which also alters the ligand geometry. The clusters’ core, water molecules, and ligand sizes are not proportional to real equivalents. The inset shows the photographs of vials before and after phase transfer from the aqueous phase to organic phase (water to chloroform) for GNCs capped with a 12-crown-4 ligand. Photos were taken within a 6 h time interval.

We design ultrasmall and amphiphilic gold nanoclusters (GNCs) stabilized with a 12-crown-4 ether and soluble in both aqueous and organic solvents. A schematic representation of our design concept is presented in Figure 1. The aqueous solution of 12-crown-4 ether-capped GNCs was prepared by gold salt reduction in the presence of a thiolated ligand, according to the protocol described in the Experimental Section. To test our hypothesis and confirm that GNCs inherit the amphiphilic properties from the 12-crown-4 ether ligand, we successfully transferred the GNCs from aqueous solution into organic solvent by a phase-transfer experiment (Figure 1, inset), and subsequently, two phases were separated. The as-prepared GNCs dispersed in water (denoted as Aq-GNCs) and transferred to chloroform (denoted as Ch-GNCs) were further characterized.

**Figure 2:**
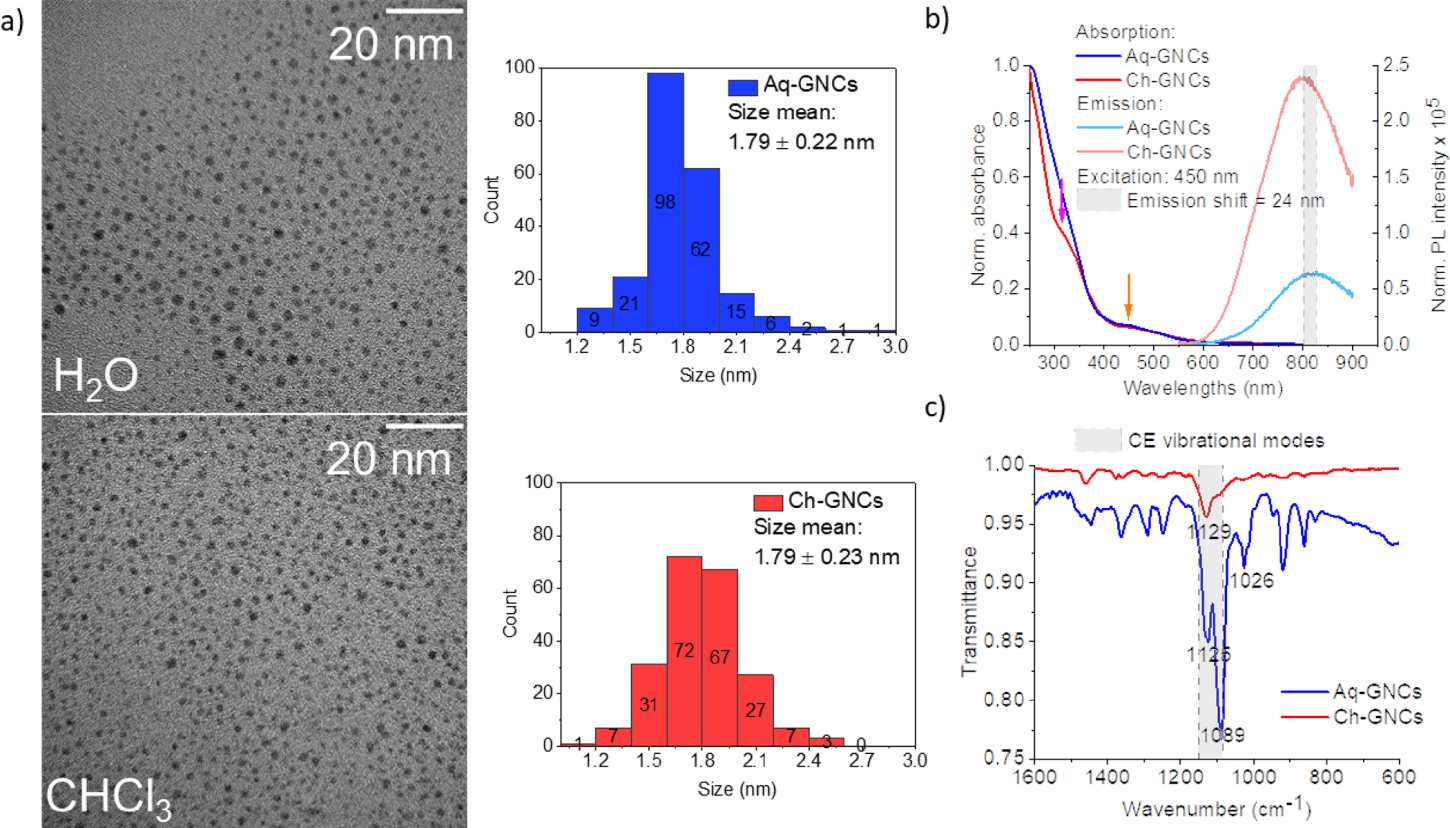
(a) TEM images of Aq-GNCs and Ch-GNCs and cluster size distribution (*n* = 215 for both TEM images), (b) normalized absorption (to 0–1) and emission (divided by corresponding absorbances at the excitation wavelength, i.e., 450 nm) of Aq-GNCs (blue spectra lines) and Ch-GNCs (red spectra lines) (magenta and orange arrows indicate distinctive bands at 320 and 450, respectively), and (c) the IR spectra recorded for Aq- and Ch-GNCs.

To probe the GNCs’ structural integrity and examine potential solvent-induced chemical reconstructions, their size distribution was estimated from transmission electron microscopy (TEM) images (Figure 2a) to be in the range of 1.79 ± 0.22 nm and 1.79 ± 0.23 nm for Aq-GNCs and Ch-GNCs, respectively (determined for *n* = 215 individual clusters).

The optical properties of GNCs in both solvents were measured (Figure 2b). Both GNC solutions are characterized by a similar absorption spectrum with a common band at 450 nm (denoted with an orange arrow). However, GNCs in aprotic solvent (CHCl_3_) are characterized by a distinct band at 320 nm (denoted with magenta arrow), which is not detected for the sample in the protic environment (H_2_O). Both GNC solutions exhibit NIR fluorescence with emission band maximums at 826 and 802 nm for Aq-GNCs and Ch-GNCs, respectively (with a 24 nm shift). To further investigate the amphiphilic properties and stability of CE-capped GNCs, a full cycle of phase transfer (from water to chloroform(I), vice versa (II), and to chloroform again (III)) was performed and the GNC solutions’ absorbance ratio (320 nm/450 nm) was monitored (see Figure S1), as described in the Experimental Section. GNCs were stable in both solvents as the absorbance ratio (equal to 4.76 and 3.87 for Aq-GNCs and Ch-GNCs, respectively) was reproduced upon a full phase-transfer cycle.

The crown ether-capped GNCs present a stable absorption and emission despite the full cycle of processing between protic and aprotic solvents, although several differences exist between the spectra in organic and aqueous solutions. The observed absorption change at 320 nm may have arisen due to the distinct crown-ether ligand conformation induced by a protic or aprotic environment as the cluster size is maintained. The study based on the native crown ethers (including 12-crown-4) conducted by Pérez et al.^44^ and Gámez et al.^45^ demonstrated that the flexibility of macrocyclic structures gives rise to multiple crown-ether conformers and that the lowest-energy conformation adopted may depend on the presence of solvents or ions in the local environment. The 12-crown-4 ligand conformation may also change in a protic or aprotic environment even while being covalently bound to gold. Such a behavior can modify the electronic structure of the entire cluster, and thus it explains the 24 nm emission shift and aforementioned absorbance change. Our hypothesis is further supported by the fact that absorbance changes induced via aggregation and structural changes of gold nanoclusters usually shift multiple bands because the entire electronic structure of the cluster system is sensitive even for a single gold atom in the range of the aurophilic interactions.^11,46^ In our case, the band located at 450 nm remains unaltered in both solvents.

Figure 2c shows FT-IR spectra with distinct peaks in the crown-ether vibrational modes’ wavenumber range, namely, at 1125 and 1089 cm^–1^ (corresponding to C–O stretching) for the hydrated sample (i.e., Aq-GNCs). These peaks are not present for Ch-GNCs of which a single peak at 1129 cm^–1^ is observed. Additionally, the peak at 1026 cm^–1^ registered for Aq-GNCs can be assigned to O–H deformation. Broad FT-IR spectra of pure solvents and Aq-GNCs as well as Ch-GNCs at the 4000–500 cm^–1^ wavenumber range are available in Figure S2a,b. However, for Aq-GNCs, a 35 cm^–1^ peak shift, which may correspond to O–H stretching (in the 3800–2800 cm^–1^ wavenumber range) was observed in comparison to pure solvent spectra (see Figure S2a). FTIR spectra registered in the protic environment differ from those of the aprotic one with both in the 1000–1200 and 3800–2800 cm^–1^ range where ether vibrational modes (e.g., C–O stretching) and O–H stretching can be detected, respectively. Therefore, our ligand may adapt different conformations by interacting with water molecules in the aqueous (protic) environment in comparison to a water-free (aprotic) environment (i.e., chloroform solvent). Our results are in good agreement with data presented in the literature where hydration shells were described via FT-IR studies on bare crown ethers^47^ and bands assigned to O–H stretching were broadened and shifted upon the intramolecular interaction of crown ethers and water molecules.

We measured the fluorescence QY of our nanoclusters using a reference dye (styryl 9 M), according to the method described by Rurack and Spieles^48^ As crown ether-capped GNCs were transferred from the aqueous to organic solvent, we observed the QY increase from 0.93 to 7.03%. It is well established in the literature that the photoluminescence properties of GNCs can be altered by ligands, which highly influence the radiative and non-radiative relaxation pathways.^49−51^ As the surface Au–S geometry of the GNCs has a profound impact on the non-radiative decay, the ligands can also exhibit different rigidities, which can significantly enhance the QY of the GNC kernel.^52^ Based on the FTIR additional peak in the C–O stretching region (1089 cm^–1^) observed solely for Aq-GNCs, we suggest that the 12-crown-4 adapts a more folded conformation to interact with a water molecule, thus altering the electronic structure of the entire cluster.

Taking advantage of the photoluminescence properties and amphiphilic character of our clusters, we applied them as markers of amyloid fibrils in a form of spherulites. Spherulites were chosen as a model of a bio-interface, which is characterized by the occurrence of hydrophobic domains. Spherulites are structurally characterized by an amorphous core (rich in α-helices) and radially growing fibrillar structures (characterized by β-sheets)—corona.^39^ As they are composed of amyloids, spherulites are characterized by a complex mix of hydrophobic and hydrophilic domains, which limits their susceptibility to staining by hydrophilic dyes, as proven experimentally.^53^

**Figure 3:**
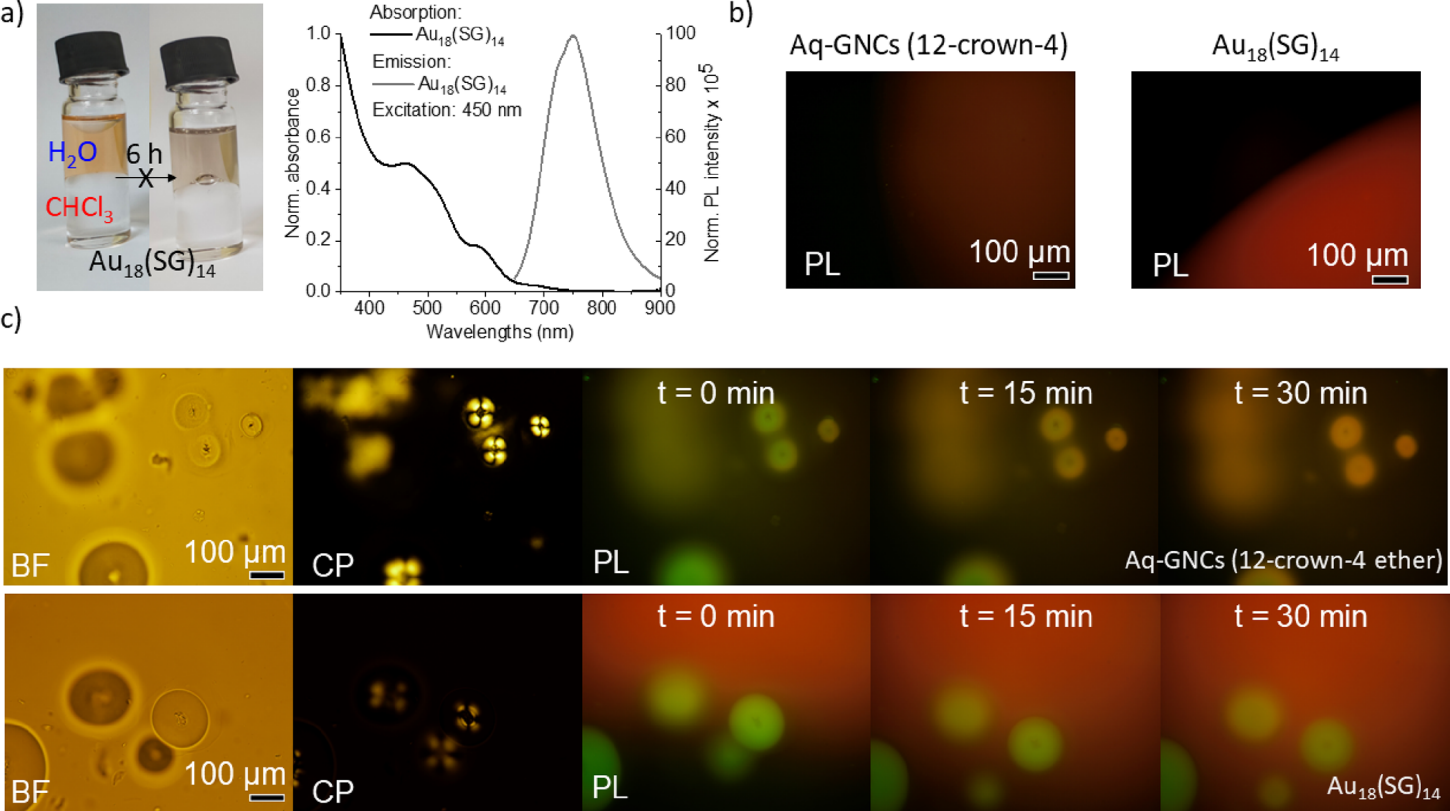
(a) Non-occurring phase transfer (water to chloroform) of Au_18_(SG)_14_ stabilized with hydrophilic ligand (SG - glutathione) and Au_18_(SG)_14_ GNCs’ optical properties, (b) fluorescence microscopy images of an aqueous solution droplet of Aq-GNCs (12-crown-4) and Au_18_(SG)_14_, (c) corresponding bright-field optical microscopy images of spherulites mixed with 12-crown-4-capped GNCs (upper panel) and Au_18_(SG)_14_ GNCs (lower panel) taken without polarizers (BF), with crossed polarizers (CP), and in the epifluorescence mode (red photoluminescence) (PL). Photos in the PL mode were taken within a 30 min period (every 15 min), starting from GNC solution addition.

Insulin spherulites were prepared (see the Experimental Section), and later on (post growth process), we stained them by mixing with GNCs. In order to compare the response of the crown-ether-stabilized clusters with a control sample stabilized with a hydrophilic ligand, we prepared water-soluble Au_18_(SG)_14_ GNCs (SG: glutathione) as described in the Experimental Section. Their absorption and emission spectra in water were measured for Au_18_ GNCs and is in a good agreement with data presented in literature.^54^ Moreover, the phase-transfer experiment with glutathione-stabilized GNCs confirmed that they do not transfer into the organic phase (Figure 3a). Both cluster solutions of Aq-GNCs (12-crown-4) and Au_18_(SG)_14_ were imaged under a microscope in the epifluorescence mode (PL), and red luminescence in both cases has been recorded (Figure 3b). The emission registered for Aq-GNCs (12-crown-4) was darker when compared to the Au_18_(SG)_14_ solution. It can be explained by the limitations of camera sensitivity in the range of the Aq-GNC (12-crown-4) emission, which is equal to 826–802 nm.

Hybrid samples were imaged under a microscope in the bright-field mode (BF), in polarized light with crossed polarizers (CP), and in the epifluorescence mode (PL) (Figure 3c). Photos in the epifluorescence mode were taken immediately after GNC addition (*t* = 0) and then after 15 and 30 min. A characteristic extinction pattern, that is, a Maltese cross, was observed with crossed polarizers (CP). It arose due to the radially growing and aligned fibrils forming the superstructure’s corona;^40^ thus, the spherulites presence was confirmed. As observed in the epifluorescence mode (PL), the spherulites glow green due to the scattering of excitation light, but a red emission from GNCs was also clearly visible. Aq-GNCs’ (12-crown-4) red emission could be registered immediately after cluster addition from entire superstructures (Figure 3c, upper panel). Majority of the Aq-GNCs (12-crown-4) in the solution assembled within the complex and densely packed amyloid superstructure. On the other hand, Au_18_(SG)_14_ GNCs did not penetrate superstructures in the presented time scale under the same experimental conditions as a red emission was not registered from spherulites’ internal areas (Figure 3c, lower panel).

Amyloid spherulites have been already identified as a host for hydrophobic and/or hydrophilic molecules (e.g., Alexa 647 (hydrophilic) and 1-anilinonaphthalene-8-sulfonic acid ANS (hydrophobic)); however, hydrophilic dyes are proven to not fully stain mature spherulites in a water/HCl mixture.^53^ Therefore, our observations regarding GNCs’ ligand hydrophobicity/hydrophilicity are in good agreement with the results presented for dyes. However, in addition to hydrophobic contacts between markers and amyloids, it has recently been reported experimentally and theoretically that 12-crown-4 ether molecules are able to interact with Aβ40 fibrils by utilizing both hydrogen bonding/electrostatic interactions with hydrophilic groups and van der Waals/hydrophobic interactions with hydrophobic residues. We hypothesize that the interaction between Aq-GNCs (12-crown-4) and the amyloid surface presented in our studies may be explained by such interactions (model presented in Figure 1 where water molecules’ solvating crown-ether ligands are released when interacting with hydrophobic groups).^32,34,55^

**Figure 4:**
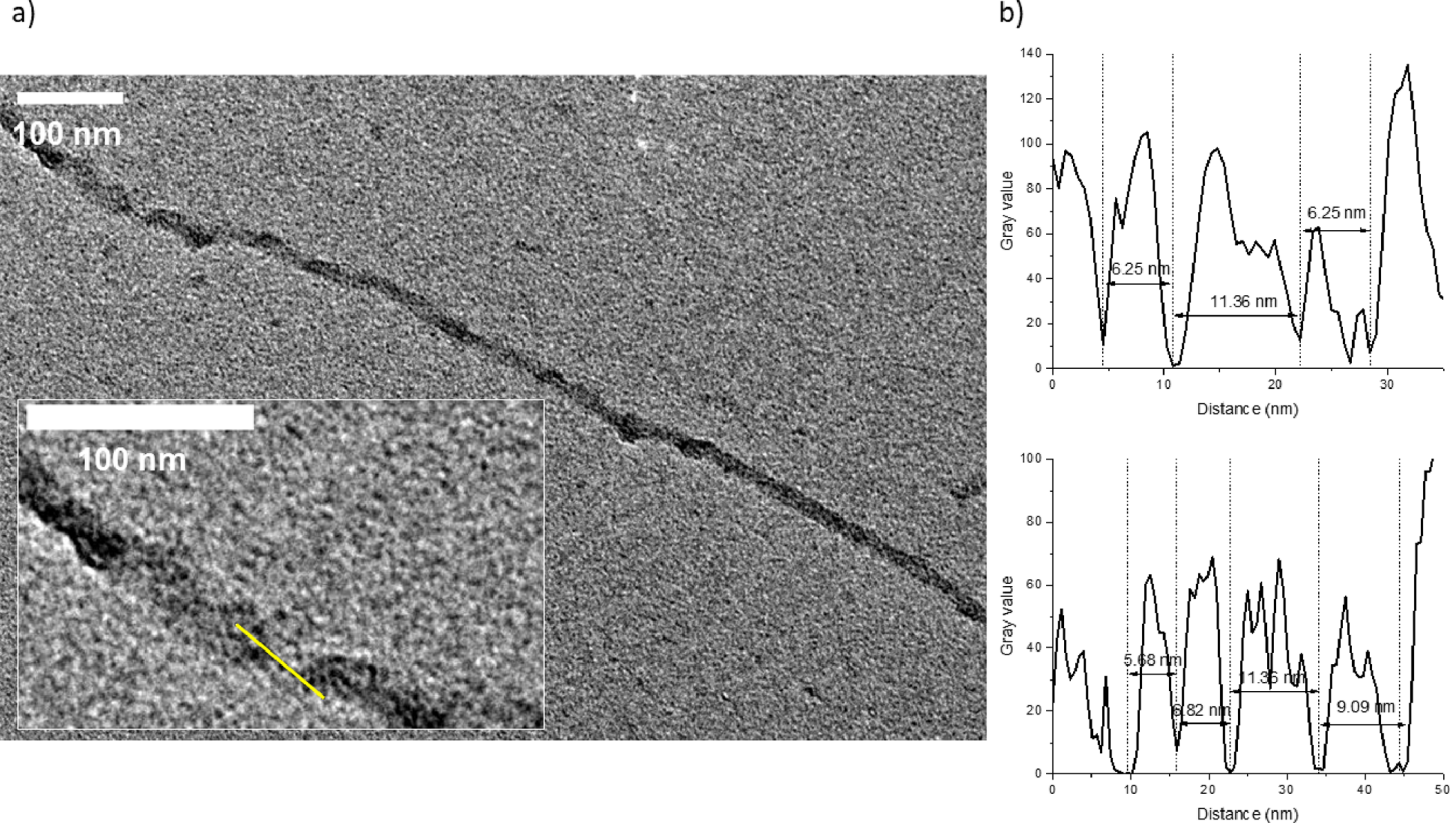
(a) High-magnification TEM image of a single amyloid fibril decorated with Aq-GNCs (12-crown-4) with an inset showing GNC organization; (b) exemplary amyloid fibril longitudinal gray scale intensity profile measured from TEM images corresponding to the GNCs’ occurrence periodicity.

As 12-crown-4-stabilized GNCs are electron-dense materials, they can be also applied in TEM imaging. Figure 4a presents the amyloid fibril decorated with our GNCs imaged with TEM. Our GNCs possess high affinity to individual amyloid fibrils as majority of the GNCs are located onto fibrillar structures with almost no clusters in the background. Our clusters are deposited on amyloid fibrils forming patterns, allowing the unraveling of the morphological features of the fibril, that is, its helical twist. Such a feature is challenging to observe by TEM imaging performed on corresponding bare and unstained amyloids (see Figure S3). Upon analysis of longitudinal and transverse profiles of single amyloid fibrils stained with GNCs from TEM images’ gray scale value, the fibril mean width and half of a helix pitch was measured to be equal to 24.34 ± 2.87 and 55.15 ± 5.62 nm, respectively. Additional profiles included in the half of a helix pitch and width measurements as well as TEM images are available in Figure S4. A corresponding solution containing amyloid fibrils was also drop-casted onto mica, dried, and imaged with an atomic force microscope (AFM; see Figure S5). Height profile analyses allowed for performing structural characterization of amyloid fibrils. The mean width and the half of a helix pitch values were calculated to be equal to 28.22 ± 1.73 and 55.96 ± 8.26 nm, respectively, which correspond to values observed with GNC-decorated fibrils under TEM (Figure 4b). The GNC occurrence periodicity on the fibrils is repetitive as they are localized every 5–10 nm in straight lines (Figure 4b) onto fibrils. Moreover, we observed that dark spots on TEM images (assigned to the highest cluster density) are also periodically repeating, which may have arisen due to the protofilaments’ twist, which introduces steric anchors for clusters to aggregate. Apart from geometrical constraints, the hydrophobic domains of the insulin amyloids may also be more exposed or occurring more frequently near the helical twist.

In insulin fibrils, hydrophobic and mixed (partially hydrophobic and hydrophilic) domains were shown to occur alternatively every ∼5 or ∼10 nm at the 24 nm segment derived from the inner part of the fibril, as was reported by Deckert-Gauding et al..^56^ Our results correspond well with the spatial distribution presented in this work. As presented theoretically^57^ and experimentally,^58^ gold nanoparticles’ affinity to amyloids is governed by hydrophilic and hydrophobic contacts. The small sizes of our clusters allow not only a reveal of the morphological features of the amyloid fibrils with a nanoscale resolution but also a discussion of their surface properties based on the cluster organization. Our results show that amphiphilic clusters can decorate fibrils in a repetitive manner and reveal their structural properties at two distinct levels: hydrophobic domain localization and twist detection. Nevertheless, further studies are necessary to fully investigate the correlation between the amphiphilic clusters’ pattering and amyloid fibrils’ structure as longitudinal and transverse surface hydrophobicity across entire fibrils has not been yet resolved.

## Conclusions

We show that gold nanoclusters capped with a 12-crown-4 ether are promising near-infrared emitting markers designed for bioimaging. Their amphiphilic properties inherited from the ligand system allow sufficient binding to biomolecules. Our clusters can be repetitively transferred between solvents, and their size, as seen by TEM imaging, remains unmodified. FTIR studies confirmed that our ligand is sensitive to its environment (i.e., protic and aprotic solvent) and can adapt certain conformations, which is also maintained for the entire cluster system. Therefore, the supramolecular functionality of crown ether ligands is preserved within our ultrasmall nanoclusters. However, their optical properties change due to altering ligand conformations upon an interaction with protic and aprotic solvents, which opens new opportunities in studies aimed at better understanding of ligands’ influence on nanocluster luminescence.

The GNCs size and physicochemical properties allow them to penetrate into complex and dense biostructures and be imaged under fluorescence microscopy as we have shown for amyloid spherulites. As gold is an electron-dense material, our GNCs are also an excellent probe for TEM bioimaging where their ultrasmall size is beneficial if a high spatial resolution is desired. Amyloid fibrils were well decorated by our GNCs, which revealed the helical organization and surface properties of the fibrils imaged under TEM. To summarize, our GNCs are a versatile tool for investigation of biological systems in a multimodal manner. We presented that the supramolecular ligand properties are transferred to and maintained in our ultrasmall nanoparticles. Ligand amphiphilicity, supramolecular interactions with a hydrogen-bond network, and electrostatic interactions allowed them to bind to amyloid fibrils in an efficient manner. Our system provides valuable insight into the manipulation of physicochemical properties of GNCs, encouraging the application of nanocluster probes in imaging and biomedical applications.

## Experimental Section

### Chemicals

Gold(III) chloride trihydrate (HAuCl_4_ × 3H_2_O, ≥ 99.9% trace metals basis), sodium borohydride (NaBH_4_, ≥ 98.0%), hydrochloric acid (HCl, 37%, ACS reagent), insulin from bovine pancreas (≥25 units/mg (HPLC), I5500), Styryl 9 M (dye content of ∼96%), l-glutathione (reduced (GSH) ≥ 98.0%), sodium cyanoborohydride (NaBH_3_CN, 95%), methanol (CH_3_OH, suitable for HPLC, ≥99.9%), hexane (CH_3_(CH_2_)_4_CH_3_, suitable for HPLC, ≥95%), isopropanol ((CH_3_)_2_CHOH, suitable for HPLC, 99.9%), diethyl ether ((CH_3_CH_2_)_2_O, contains 1 ppm BHT as inhibitor, anhydrous, ≥99.7%), and chloroform (CH_3_Cl, suitable for HPLC, ≥99.8%, contains 0.5–1.0% ethanol as a stabilizer) were used as purchased from Sigma-Aldrich. 2-(Mercaptomethyl)-12-crown-4 was purchased from Prochimia Surfaces. High-purity water (Milli-Q quality) was used throughout all the experiments.

### Gold Nanocluster Synthesis (12-Crown-4)

Ultrasmall 1.78 nm Au/12-crown-4-SH GNCs were synthesized based on the method described by Grzelczak et al.^28^ A mixture of a 1:3 HAuCl_4_ × 3H_2_O:2-(mercaptomethyl)-12-crown-4 molar ratio was used. Briefly, 2.5 mL of a 3 mM solution of gold(III) chloride trihydrate in methanol was added to 2.5 mL of 9 mM solution of 12-crown-4 in methanol and kept stirred (1000 rpm) in a 20 mL scintillator vial (27.3 × 60 mm, BIONOVO) for 1 h in an ice bath. Then, 0.4 mL of freshly prepared and ice-cold 0.1 M NaBH_4_ methanolic solution was rapidly injected under vigorous stirring (1300 rpm) into the gold:capping agent mixture, which resulted in a quick color change—from pale and cloudy yellow to deep brown. Then, the solution was left under gentle stirring for 1 h for NaBH_4_ hydrolysis to be completed. Next, the resulting 12-crown-4-capped-GNC dispersion was transferred with a glass Pasteur pipette to a 50 mL round-bottom flask and rotary-evaporated at 30 °C. The dry residue was washed three times with 30 mL of hexane, dried, and again washed three times with the same amount of diethyl ether and left for 30 min at ambient temperature for solvent residues to evaporate. Next, the resulting dry powder was redispersed in 5 mL of isopropanol and filtered off with a syringe filter (0.22 μm pore size, 13 mm diameter, Millex-GP PES Millipore Express membrane, hydrophilic, Sigma-Aldrich). The filtered solution was rotary evaporated at 30 °C, and the resulting purified dry residue was dispersed in 10 mL of high-purity water (Milli-Q).

### Gold Nanocluster Synthesis (Au_18_(SG)_14_)

Glutathione-stabilized gold nanoclusters were synthesized based on the combined protocols by Ghosh et al.,^59^ Yang et al.,^60^ and Stamplecoskie et al.^61^ Briefly, 150 mg of GHS was dissolved in 0.6 mL of MeOH and 0.6 mL of water in a 20 mL round-bottom flask. After 10 min, a 0.3 mL aliquot of 0.636 M gold(III) chloride trihydrate was added and left until the solution became colorless. In the next step, the solution was diluted to 15 mL with methanol, which was subsequently followed by dropwise addition of 2.25 mL of NaBH_3_CN (220 mM) under vigorous stirring. After a few hours, the precipitate was collected from centrifugal precipitation (10 min, 700 rcf) and washed with methanol several times. The product was rotary-evaporated at 30 °C, and the resulting purified dry residue (red powder) was dispersed in 2 mL of high-purity water (Milli-Q).

### Gold Nanocluster Phase Transfer and Their Reversibility

A 1 mL aliquot of as-synthesized aqueous GNC solution was thoroughly mixed with the same volume of organic solvent, that is, chloroform, and left undisturbed for 6 h for emulsion to destabilize, phases to separate, and clusters to diffuse. After that, the clear aqueous phase was discarded and the remaining organic phase containing GNCs was characterized using TEM imaging and UV–vis spectroscopy. To test the reversibility of the process, GNCs in chloroform were rotary-evaporated at 30 °C and the resulting dry residue was redispersed in water, tested in UV–vis spectroscopy, and again transferred to the chloroform by phase transfer, as described above.

### UV–Vis Spectroscopy

The UV–vis spectra of organic and aqueous solutions with corresponding concentrations of GNCs were measured in a QS high-precision cell (10 mm, Hellma Analytics) using a JASCO V-670 spectrophotometer and Edinburgh Instruments FLS100 spectrofluorometer. The fluorescence QY was calculated using the reference dye (styryl 9 M) according to the method described by Rurack and Spieles^48^ (excitation wavelength was set to 450 nm).

### Insulin Amyloid Incubation

Amyloid fibrils were prepared by dissolving 10 mg of bovine insulin powder in 1 mL of Milli-Q water with the pH adjusted to 2 (with hydrochloric acid). In the next step, the solution was incubated at 70 °C for 24 h and constantly stirred at a rate of 700 rpm (in an Eppendorf Thermomixer C). Amyloid spherulites were prepared in the same manner without mixing. All incubations were conducted in 1.5 mL Eppendorf Safe-Lock Tubes (polypropylene) sealed with PTFE thread seal tape (12 m × 12 mm × 0.1 mm, 60 gm^2^). As incubation was finished, solutions containing amyloid fibrils and spherulites were diluted with high-purity water (Milli-Q) to the final concentrations of 0.05 and 0.5 mg/mL, respectively.

### AFM Imaging and Sample Preparation

A 100 μL aliquot of amyloid fibril solutions (0.05 mg/mL) was mixed with 100 μL of GNC stock solution and left undisturbed in 5 °C for 6 h to let GNCs diffuse onto amyloid fibrils. After that, the solution was pipetted onto a mica surface (V-1 Quality, 15 mm × 15 mm, Sigma-Aldrich) and left for 1 min under ambient conditions. In the next step, mica surfaces were washed thoroughly with 5 mL of high-purity water and left to dry. As prepared, samples were imaged using a Dimension V Veeco AFM instrument in the tapping mode. Morphological analysis of fibrils was performed using Nanoscope Software 7.30.

### TEM Imaging and Sample Preparation

A 100 μL aliquot of amyloid fibril solutions (0.05 mg/mL) was mixed with 100 μL of GNC stock solution and left undisturbed at 5 °C for 6 h to let GNCs diffuse onto amyloid fibrils. After that, a 10 μL aliquot of stained fibrils was pipetted on a plasma-cleaned (EMITECH, K1050X) copper grid with carbon only support film (AgarScientific, AGG2200) and left undisturbed for 5 min for fibrils and clusters to deposit onto a grid surface. Then, to avoid a thick film formation, the grid was subsequently dipped into Eppendorf Safe-Lock Tubes filled with 2 mL of water and methanol. As-cleaned samples were left to dry under ambient conditions prior to imaging. Then, the images were taken using a a JEOL F200 S/TEM microscope with an accelerating voltage of 200 kV.

### Fluorescence, Bright Field, and Polarized-Light Microscopy Imaging

A 50 μL aliquot of amyloid spherulite solution (0.5 mg/mL) was pipetted onto a microscope slide with a well (Super White Glass, 76 × 26 × 1 mm, CHEMLAND). After that, a 50 μL aliquot of GNC stock solution (Au_18_(SG)_14_ or Au/12-crown-4) was added. Then, samples were covered with a coverslip and immediately measured in the chosen timescale (*t* = 0, 15, and 30 min). The samples were imaged under an Olympus BX60 optical microscope to obtain bright-field, polarized-light, and fluorescence images using an Olympus UPlanFLN 20×/0.5 NA objective. Fluorescence imaging was performed in a wide-field epifluorescence mode, and excitation wavelengths were set in the 460–495 nm range.

## Supporting Information Available

Reversibility of the phase transfer processing test (UV–vis), FT-IR spectra of clusters and pure solvents, TEM image of the unstained amyloid fibril, TEM image and morphology analysis of the stained amyloid fibril, and AFM image and morphology analysis of the unstained amyloid fibril (PDF)

## Abbreviations

GNCs: gold nanoclusters
Aq: GNCs’ aquatic solution of gold nanoclusters
Ch-GNCs: organic solution (chloroform) of gold nanoclusters
PD: Parkinson’s disease
AD: Alzheimer’s disease
TERS: tip-enhanced Raman spectroscopy
TEM: transmission electron microscopy
AFM: atomic force microscopy
QY: photoluminescence quantum yield
FTIR: Fourier-transform infrared spectroscopy
BF: bright-field optical microscopy
CP: optical microscopy with crossed polarizers
PL: fluorescence microscopy (in the epifluorescence mode)
GSH: glutathione
