# Supplementary material for: Crown Ether-Capped Gold Nanoclusters as a Multimodal Platform for Bioimaging
Source: ACS Omega. 2023 Mar 13;8(12):11503–11. doi: 10.1021/acsomega.3c00426 (PMC10061685; doi:10.1021/acsomega.3c00426)
Supplement: Supplementary file 1 — ao3c00426_si_001.pdf [file ao3c00426_si_001.pdf]

Crown ether capped gold nanoclusters as a multimodal platform for bioimaging - **SI**

Patryk Obstarczyk[1], *Anna Pniakowska*[1], *Nonappa*[2], *Marcin P. Grzelczak*[1], *Joanna Olesiak-Bańska*[*1]

[1]Institute of Advanced Materials, Wroclaw University of Science and Technology, Wrocław, Poland

[2]Faculty of Engineering and Natural Sciences, Tampere University, FI-33720, Tampere, Finland

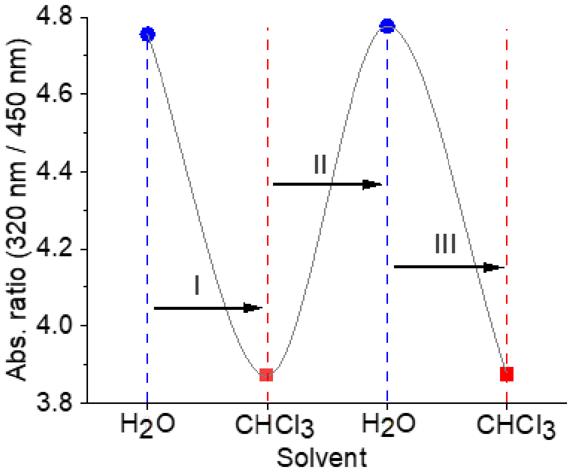

Figure S1. *Reversibility of the phase transfer process monitored by the sample absorbance ratio (Abs at 320 nm/Abs at 450 nm) measured alternately in a protic and aprotic solvent (from H2O to CHCl3 (I), vice versa (II) and to CHCl3 again (III).*

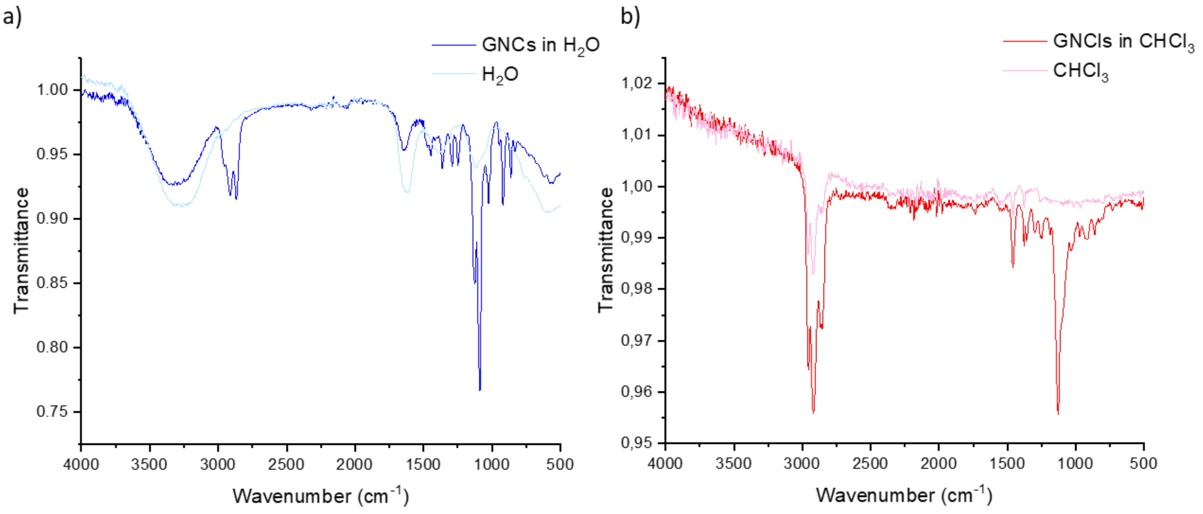

Figure S2. Broad FT-IR spectra of pure solvents and Aq-GNCS as well as Ch-GNCs at 4000 – 500 cm-1 wavenumber range.

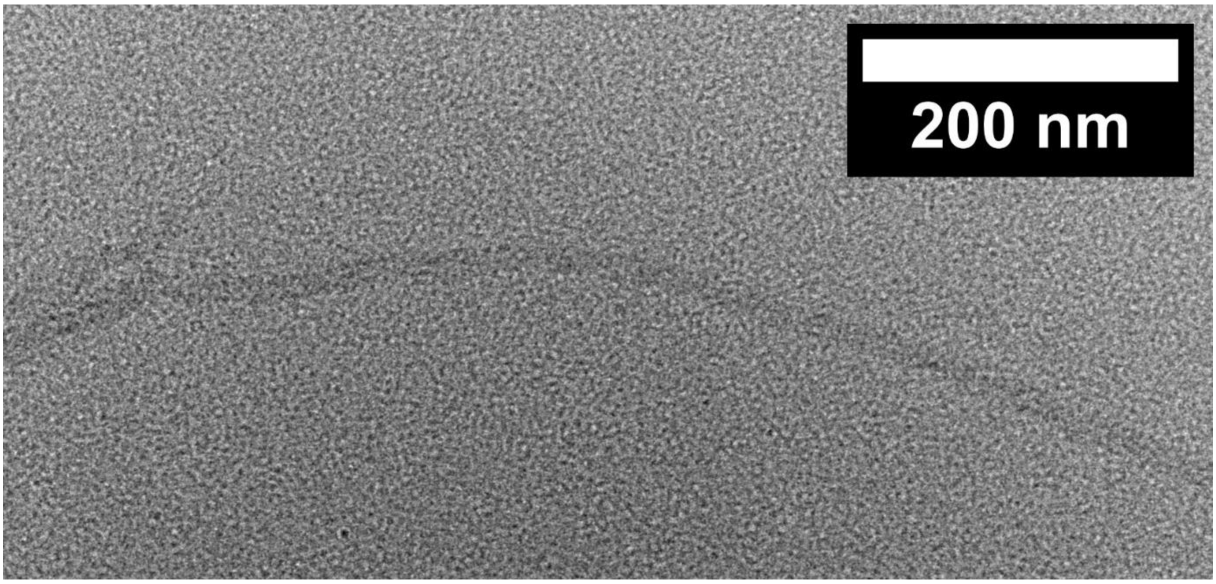

Figure S3. *Bare (unstained) amyloid fibril.*

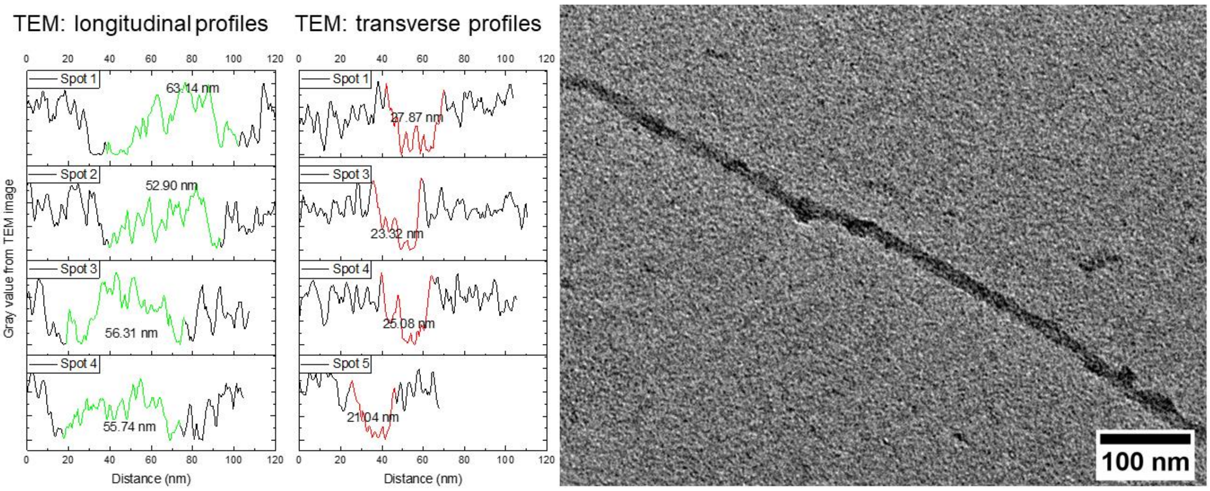

Figure S4. Longitudinal and transverse grey scale profiles taken from TEM image of amyloid fibril (decorated with Au/12-crown-4 clusters).

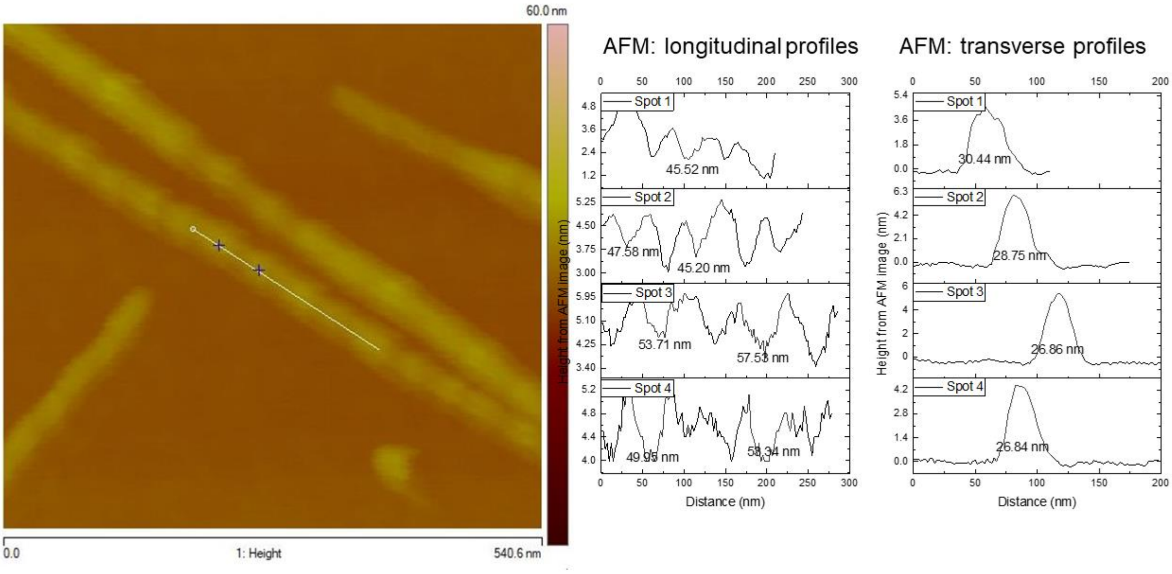

Figure S5. AFM image of amyloids deposited on mica: longitudinal and transverse height profile from AFM image.
